# Changes in health behaviours during the COVID-19 pandemic and effect on weight and obesity among older people in England

**DOI:** 10.1038/s41598-023-41391-z

**Published:** 2023-09-05

**Authors:** Jingmin Zhu, Giorgio Di Gessa, Paola Zaninotto

**Affiliations:** Department of Epidemiology and Public Health, University College London, 1-19 Torrington Place, London, WC1E 7HB UK

**Keywords:** Weight management, Risk factors, Epidemiology

## Abstract

During COVID-19 lockdown, negative changes in health behaviours have been reported in European older adults. However, less is known about the consequences of these changes on weight gain and obesity, especially in older adults living in England. This study explored the association of health behaviour changes with weight and obesity in English older adults aged 50 years and older, during lockdowns in 2020. We included 4182 participants of the English Longitudinal Study of Ageing COVID-19 sub-study in June/July and Nov/Dec 2020 who also had pre-pandemic data. Perceived changes in health behaviours were regressed on weight and obesity, adjusted for pre-pandemic weight or obesity, and several covariates. Results suggested that less exercise, more sedentariness, eating more and alcohol drinking were associated with a significant increase in weight at both timepoints. Meanwhile, less sedentariness and eating less significantly reduced weight in Nov/Dec 2020. A higher risk of obesity at both timepoints was found in adults sitting, eating, or sleeping more than usual. To conclude, during UK lockdown, older people who engaged in risky health behaviours were at higher risks of weight gain and obesity both in the short run and long term. Considering potential health risks associated with obesity and disruptions in routine lifestyle in the older population even after the pandemic, improved weight management interventions are necessary nationwide.

## Introduction

Since the outbreak of COVID-19, social restrictions such as working from home and self-isolation have been widely implemented to diminish the chances of infection, especially among older people who are more vulnerable to coronavirus^1^. People’s access to outdoor activities was restricted. Some people experienced drastic changes in daily routine, and some suffered from psychological distress or anxiety during the lockdown^2–5^. As a result, negative changes in health behaviours have occurred among older populations worldwide^6^. A lower level of physical activity among older adults was found in the UK^7,8^, Italy^2,9–11^, India^5^, Japan^12^, Germany^13^, and China^14^. Reports have shown that more time was spent on sedentary activities^5,10^ and on screen^14^. Worse sleep and unusual sleeping patterns were also reported among older people^2,5^.

Furthermore, weight gain has been consistently reported during the pandemic^3,6,15,16^. As shown by some studies, there could be a higher risk of weight gain and obesity in the older population. For example, Lithuanian people older than 50 years had 1.80 times higher odds of gaining weight during lockdown than those adults younger than 36 years^15^. One month after the national lockdown, Italian adults older than 50 years experienced an increase of 1.8 kg in weight and an increase of 0.7 kg/m^2^ in Body Mass Index (BMI), compared to 1.24 kg and 0.47 kg/m^2^ in adults aged 50 and below^16^. The increasing risk of obesity in older populations is detrimental considering its association with a higher risk of morbidity, incidence of cardiovascular disease, mortality, disability and cognitive impairment in the old population^17–21^.

Few studies documented changes in body weight during the COVID-19 lockdown in the UK. The mean BMI of 206 adults aged 18 and older living in Aberdeen (Scotland) increased from 25.8 kg/m^2^ before the COVID-19 lockdown to 25.9 kg/m^2^ over the months from April to June 2020 during lockdown^22^. Over 750,000 English people, more than 90% of whom aged 40 and older, engaged in the English NHS Diabetes Prevention Programme during April 2020 and March 2021 had 0.4 kg/m^2^ higher BMI and 2.4 kg higher body weight on average compared to those entering NHS Diabetes Prevention Programme over a 3-year period ahead of UK COVID-19 lockdown (April 2017 to March 2020)^23^.

The relationship between health behaviours and obesity is well-established in the literature^24–27^. More frequent physical activity, better sleep and no smoking significantly reduced the risk of obesity in European older population older than 65 years^24^. Lack of physical activity increased the BMI of German old people aged 79 and above^25^. In Canadian old adults aged 65 and older, lack of physical activity and heavy alcohol consumption were significant predictors of obesity^26^. A systematic review showed that alcohol intake was positively associated with weight gain in heavy drinkers^27^.

In recent studies, the association between health behaviours and body weight has been examined in the context of the COVID-19 pandemic^3,15,16,28–30^. Changes in health behaviours and weight were usually collected over 1 month to 6 weeks after the lockdown. A cross-sectional analysis was conducted to examine the association between changes in health behaviours and changes in weight or BMI in all adults. Only one study provided evidence on people aged 55 and over and found that physical activity, eating more, and increased alcohol drinking were all significant risk factors of weight gain^28^.

Therefore, there still exists a gap in the literature on the impact of changes in health behaviours on weight and/or obesity levels among older people. Little is known about the weight and obesity of the older population during lockdown in the UK and to what extent it is attributed to changes in health behaviours. Furthermore, even though the immediate effect of health behaviour changes on body weight during lockdown has been reported, no studies so far have examined the longer-term effect. To this end, this study explores changes in health behaviours in relation to both body weight and obesity during the COVID-19 pandemic among older people aged 50 years and older living in private households in England, investigating both short- and long-run associations.

## Methods

### Study population

We used the most recent pre-pandemic data (Wave 9, collected in 2018/19) and the two waves of the COVID-19 sub-study (collected in June/July and Nov/Dec 2020 respectively) of the English Longitudinal Study of Ageing (ELSA)^31^. ELSA is a longitudinal biennial survey representative of individuals aged 50 and over in private households. During the pandemic, 9392 ELSA members were invited to participate online or by CATI (Computer-Assisted Telephone Interviewing) in the COVID-19 sub-study (75% response rate in both waves, with 94% participants of COVID-19 wave 1 successfully interviewed in COVID-19 wave 2). Prior to the pandemic, respondents of ELSA were interviewed face-to-face every two years. Analyses were based on core members and partner respondents who participated in both COVID-19 waves (N = 5827) with available information on their weight also in Wave 9 (N = 4182). Further details of the survey’s sampling frame and methodology can be found at https://www.elsa-project.ac.uk. The present study was approved by the South Central-Berkshire Research Ethics Committee (17/SC/0588), with the COVID-19 sub-study approved by the University College London Research Ethics Committee. All methods were performed in accordance with the relevant guidelines and regulations. Informed consent was obtained from all participants. All data are available through the UK Data Service (SN 8688 and 5050).

### Main measurements of interest

#### Perceived changes in health behaviours during the COVID-19 pandemic

Respondents were asked to report perceived changes in health behaviours in June/July 2020. The wording of the question was: “*Since the coronavirus outbreak began in February, please say whether you have been doing each of the following less than usual, about the same, or more than usual*” with the following activities mentioned: sitting down, watching TV, doing physical exercise, eating, and sleeping. Those who were drinking alcohol at the time of the interview were also asked the following question: “*Since the start of the coronavirus outbreak, please say whether you have been drinking less than usual, about the same, or more than usual*”. All these categorical variables were coded for analysis using “About the same” as the reference group (i.e., 0 “About the same” 1 “Less” and 2 “More”), with one more group 3 “Never” for drinking alcohol.

#### Weight, overweight, and obesity

We considered three health outcome measures of weight. At each COVID-19 wave, respondents were asked to self-report their weight in absolute values (either in kilograms or in stones and pounds, with all measurements then converted into kilograms). In Wave 9, however, weight was measured using a portable electronic scale. Respondents were asked to remove their shoes and any bulky clothing, and measurements were recorded to the nearest 0.1 kg, with respondents who weighed more than 130 kg being asked for their estimated weights because the scales are inaccurate above this level. These estimated weights, together with measured weights were included in the analysis without standardization or adjustment. At the second COVID-19 wave, all respondents were also asked to report their height without shoes (either in centimetres or feet and inches, with all height measurements then converted into centimetres). Weight change in percentage at two COVID-19 waves was further calculated with reference to pre-pandemic weight from Wave 9. Based on the respondents’ weight at each wave and assuming the height constant for all three timepoints considered, we calculated the BMI as weight in kilograms divided by the square of the height in metres (kg/m^2^). BMI is a widely accepted measure of weight for height, and in line with the World Health Organization (WHO) classification of weight status^32^, we categorised respondents as normal weight if their BMI was less than 25 kg/m^2^, as overweight if their BMI was between 25 and 30 kg/m^2^, and as with obesity if their BMI was equal to or greater than 30 kg/m^2^. We did not consider the underweight group on its own as there were too few respondents in this category (N = 45). Those underweight were grouped with normal weight.

#### Covariates

Based on the literature review, our analyses controlled for a wide range of demographic, socio-economic characteristics, health, and social support characteristics^15,25,28,33^. We controlled for age groups (50–59; 60–69; and 70+) to account for non-linear relationships with the outcome variables; sex; and ethnicity (White vs non-White participants due to data constraints in ELSA), which were measured at COVID-19 wave 1 in June/July 2020. To capture respondents’ socio-economic characteristics, we used five variables including pre-pandemic education, wealth, and occupational social class collected in Wave 9, along with living arrangements, and access to enough food measured in COVID-19 wave 1. Educational level was recoded into 0 “high” (university or above) and 1 “low” (below university), following the International Standard Classification of Education (http://www.uis.unesco.org/), consistent with the literature^15^. We categorised respondents by quintiles of wealth (total net non-pension non-housing wealth) coded as 0 “Lowest”, 1 “2nd”, 2 “3rd”, 3 “4th”, 4 “Highest”. Occupational social class was based on current or most recent job (coded as 0 “managerial and professional”; 1 “intermediate”; 2 “routine and manual occupations”; 3 “other” following the National Statistics Socio-Economic Classification). For living arrangements, we considered whether respondents were living alone (0 “no” and 1 “yes”). Finally, we considered a binary variable indicating whether respondents (and their family members) had enough kinds of food they wanted to eat always or not (coded as 0 “yes” and 1 “not always enough food”).

We also accounted for baseline health measured at COVID-19 wave 1 in June/July 2020. In particular, we controlled for anxiety, depression, self-reported quality of sleep, and cigarette smoking. Anxiety was assessed by Generalized Anxiety Disorder with 7 psychiatric items (GAD-7). Respondents with a score of ten and above were classified as having at least moderate levels of anxiety^34^, the variable was coded as 0 “no anxiety” 1 “anxiety”. Symptoms of depression were measured by an abbreviated version of the validated Centre for Epidemiologic Studies Depression (CES-D)^35^, which is used to identify people “at risk” of depression in population-based studies^36^. The scale includes 8 binary (no/yes) questions that enquire about whether respondents experienced any depressive symptoms, such as feeling sad or having restless sleep, in the week prior to the interview. We classified respondents who reported four or more depressive symptoms on the CES-D scale as with elevated depressive symptoms (coded as 0 “no depression” and 1 “elevated depressive symptoms”)^37,38^. The self-reported quality of sleep was a 5-scale indicator, recoded into a binary variable (0 “poor or fair sleep” and 1 “at least good sleep”). Cigarette smoking status was coded into 0 “not currently smoking” and 1 “current smoker”.

Finally, we considered three measures of social isolation during the first ELSA COVID-19 wave. ELSA respondents were asked questions about real-time contact (by telephone or video calling) with family outside the household and with friends in the past month. We used a binary variable coded as 1 for respondents having contact with family and friends less than once a week or never and 0 otherwise. Moreover, using the short version of the Revised UCLA loneliness scale^39^, we classified respondents as indicating greater loneliness if their scores were equal or greater than six. Shielding (0 “no” and 1 “yes”) was defined as self-isolation or staying at home either in April or one week prior to the interview in June/July 2020.

### Statistical analyses

Following descriptive analysis, we investigated the associations of perceived changes in health behaviours at COVID-19 wave 1 with weight and obesity measured at two COVID-19 waves, cross-sectionally. For weight in kilos, we used linear regression models whereas for obesity we used logistic models. Robustness checks were also carried out repeating the analyses using BMI, both as a continuous variable and as an ordinal categorical variable. Sensitivity analysis was conducted using weight changes in percentage with reference to pre-pandemic weight as an outcome. We considered each perceived change in health behaviours separately, and all models adjusted for demographic, socio-economic characteristics, health, and social isolation, as well as for pre-pandemic relevant measures of weight/obesity. Each health behaviour variable measuring perceived changes was treated as categorical in all analyses, with the category “About the same” as reference group. All analyses were performed using Stata 17. Estimates were adjusted for cross-sectional probability sampling weights (for analysis of COVID-19 wave 1 exposures and outcomes) and for longitudinal sampling weights (for analysis of COVID-19 wave 1 exposures and COVID-19 wave 2 outcomes) to account for different probabilities of being included in the sample and for nonresponse to the survey. Variance Inflation Factor (VIF) test was applied to test collinearity^40,41^.

## Results

### Baseline characteristics at COVID-19 wave 1

Table 1 describes perceived changes in health behaviours of our analytical sample at COVID-19 wave 1 in June/July 2020. A total of 4 182 participants were included. The majority of participants did not report any changes in their lifestyle behaviours during the first months of the COVID-19 pandemic. However, 40% participants reported spending more time sitting, and about one third reported watching more TV and engaging less in physical activity. About one in five participants (19%) reported eating more and 12% reported drinking more alcohol than usual. Lastly, 22% reported less sleep (and nearly half of the sample reported poor quality of sleep).

**Table 1:** Perceived changes in health behaviours of the analytical sample in June/July 2020 (N = 4182).

	N (%, weighted)
Time spent sitting	
About the same	2187 (51.5)
Less	363 (8.4)
More	1632 (40.1)
TV watching	
About the same	2418 (57.2)
Less	438 (10.6)
More	1326 (32.2)
Physical activity	
About the same	1925 (46.4)
Less	1442 (34.6)
More	815 (19.0)
Eating	
About the same	3117 (72.5)
Less	336 (8.5)
More	729 (19.0)
Sleeping	
About the same	2989 (69.1)
Less	851 (22.0)
More	342 (8.9)
Alcohol drinking	
Never	1395 (35.8)
About the same	1685 (37.8)
Less	581 (14.2)
More	521 (12.2)

Table 2 depicts baseline characteristics of our analytical sample collected at the first COVID-19 wave in June/July 2020. The average weight of 4 182 participants was 77.1 kg (Standard Deviation: 17.0) and the average BMI was about 27.4 kg/m^2^ (Standard Deviation: 5.3). There was about a quarter of participants with obesity, whereas the prevalence of overweight (not including obesity) was around 39.0%. Among those with normal weight prior to the pandemic, 8.9% reported being overweight but not with obesity, and 0.23% with obesity in Jun/July 2020; similar percentages were found in Nov/Dec 2020, with 9% being overweight, and 0.23% with obesity (see Table S1).

**Table 2:** Baseline characteristics of the analytical sample in June/July 2020 (N = 4182).

	N (%, weighted)
Weight in kg, mean (standard deviation)^a^	77.1 (17.0)
Body mass index, mean (standard deviation)	27.4 (5.3)
Body mass index categories	
Normal or underweight	1545 (35.2)
Overweight	1648 (39.0)
With obesity	989 (25.8)
Age groups	
50–59	647 (27.8)
60–69	1408 (30.3)
70+	2127 (41.9)
Women	2480 (56.1)
Non-white ethnicity	72 (3.0)
High education^b^	991 (19.9)
Wealth quintile	
Lowest (below £139,000)	771 (26.0)
2nd (£139,000–280,000)	858 (20.8)
3rd (£280,200–441,000)	841 (18.3)
4th (£441,003–730,000)	868 (18.3)
Highest (above £730,000)	844 (16.6)
Social class	
Managerial	1352 (26.2)
Intermediate	1026 (21.0)
Manual	1231 (30.8)
Other or incomplete	573 (22.0)
Living alone	957 (24.3)
Not always enough food	563 (14.8)
Anxiety	288 (8.0)
Depressed	724 (19.0)
Self-reported poor/fair sleep	1669 (41.8)
Current smoker	304 (10.4)
Loneliness	842 (22.2)
Low social contact^c^	281 (7.3)
Shielding^d^	3829 (89.5)

^a^The number of participants with weight over 130 kg is 23 at baseline in June/July 2020, 25 in Nov/Dec 2020, and 29 in Wave 9 prior to the COVID-19 pandemic.

^b^High education is defined as university degree and above.

^c^Low social contact is defined as less than weekly contact with immediate family or friends. Contact includes video calls and phone calls.

^d^Shielding is defined as self-isolation or staying at home.

### Association of health behaviours with weight and obesity

**Figure 1:**
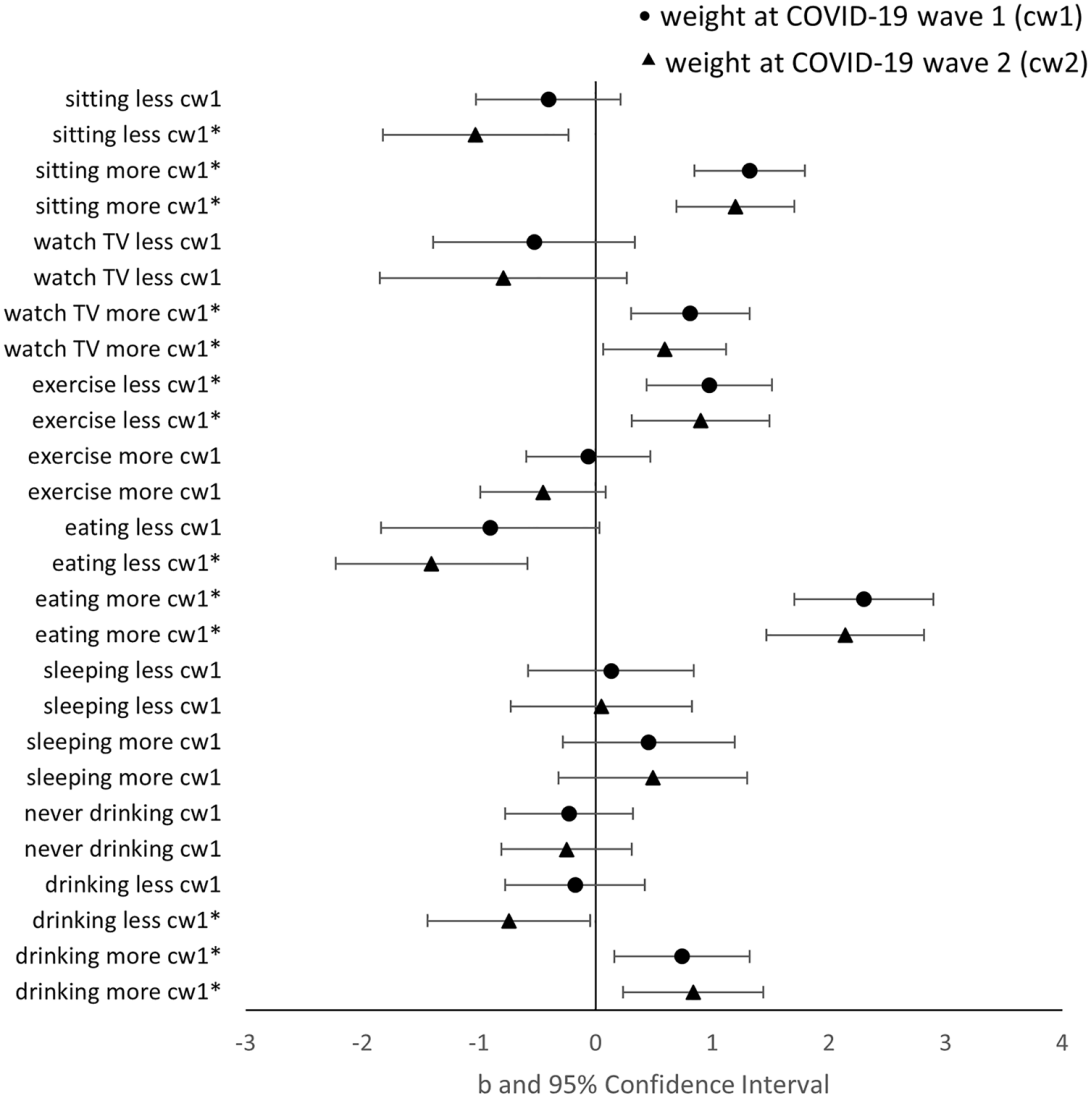
Fully adjusted associations of perceived changes in health behaviours with weight at two timepoints. N = 4182. A continuous variable, *weight*, is used as outcome. Separate linear regression models are estimated for each health behaviour with full adjustment. The reference category of each perceived change in health behaviour is “About the same”. All models show high goodness of fit (p < 0.001). Perceived changes in health behaviours are collected in COVID-19 wave 1. The outcome of weight is measured in two COVID-19 waves. cw1 and cw2 denote the timepoint at which the outcome variable is collected. cw1: COVID-19 wave 1 in June/July 2020; cw2: COVID-19 wave 2 in Nov/Dec 2020. Asterisk: significant at 95% confidence level.

**Figure 2:**
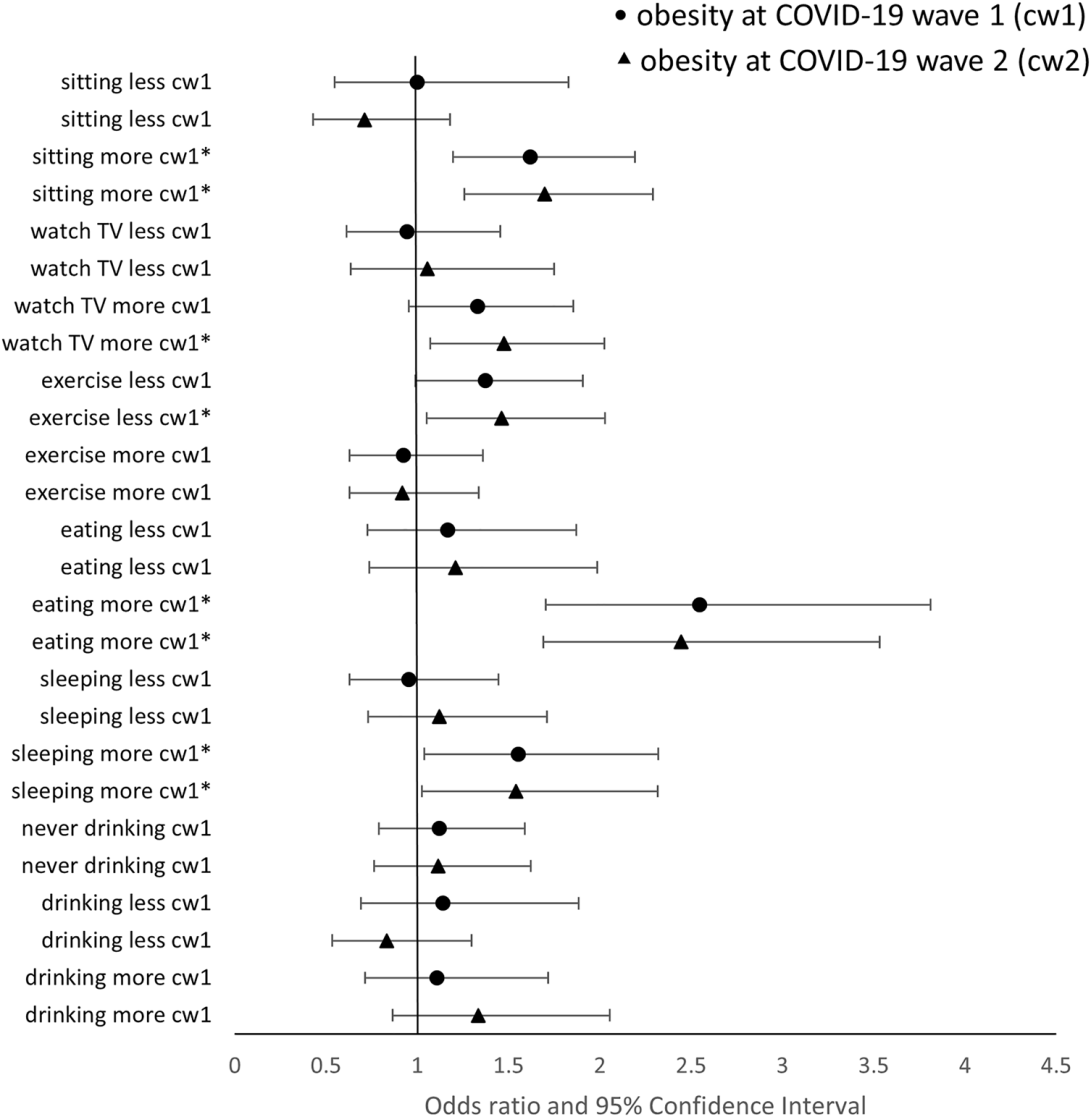
Fully adjusted associations of perceived changes in health behaviours with obesity at two timepoints. A binary variable, *obesity*, defined as 1 if BMI ≥ 30 kg/m^2^ and 0 otherwise, is used as outcome. Separate logistic regression models are estimated for each health behaviour with full adjustment. The reference category of each perceived change in health behaviour is “About the same”. All models show high goodness of fit (p < 0.001). Perceived changes in health behaviours are collected in COVID-19 wave 1. The outcome of obesity is measured in two COVID-19 waves. cw1 and cw2 denote the timepoint at which the outcome variable is collected. cw1: COVID-19 wave 1 in June/July 2020; cw2: COVID-19 wave 2 in Nov/Dec 2020. Asterisk: significant at 95% confidence level.

Fully adjusted associations of perceived changes in health behaviours in June/July 2020 with weight and obesity measured in June/July and Nov/Dec 2020 are depicted in Figs. 1 and 2, respectively. The mean value of VIF was always around 1.50, indicating a moderate correlation between independent variables^41^.

Results suggested that perceived changes in sedentary behaviours were significantly associated with higher weight and obesity. Spending more time sitting was associated with an increase in weight both in the first months of the pandemic (June/July 2020, 1.32 kg, 95% CI: 0.85–1.80, p < 0.01) and in Nov/Dec 2020 (1.20 kg, 95% CI: 0.69–1.71, p < 0.01). Compared to people not reporting any changes in sedentary behaviours, participants who spent more time sitting were also at a higher risk of obesity at both time points (June/July 2020 OR = 1.62, 95% CI: 1.19–2.19, p < 0.01; Nov/Dec 2020 OR = 1.70, 95% CI: 1.26–2.29, p < 0.01). Respondents who reported sitting less were more likely to have lost weight by Nov/Dec 2020 (−1.03 kg, 95% CI: −1.82 to −0.23, p = 0.01).

Similarly, participants who spent more time watching TV reported significantly higher weight at both time points (0.81 kg, 95% CI: 0.31–1.32, p < 0.01 in June/July 2020; 0.59 kg, 95% CI: 0.06–1.12, p = 0.03 in Nov/Dec 2020) and were more likely to be with obesity in Nov/Dec 2020 (OR = 1.47, 95% CI: 1.07–2.02, p = 0.02).

Changes in physical activity were significantly related to changes in weight. As expected, less physical activity was associated with both weight gain [0.97 kg in June/July, (95% CI: 0.44–1.51, p < 0.01) and 0.90 kg in Nov/Dec (95% CI: 0.31–1.49, p < 0.01)] and a higher risk of obesity in Nov/Dec 2020 [(OR = 1.46, 95% CI: 1.05–2.03, p = 0.02)].

Eating more was also a significant risk factor of increased weights and obesity in both June/July and Nov/Dec 2020. Compared to those having no change in eating behaviour, participants eating more were heavier [2.30 kg in June/July 2020 (95% CI: 1.70–2.89, p < 0.01) and 2.14 kg in Nov/Dec 2020 (95% CI: 1.47–2.81, p < 0.01)] and more likely to be with obesity [OR = 2.55 in June/July 2020 (95% CI: 1.70–3.81, p < 0.01) and OR = 2.44 in Nov/Dec 2020 (95% CI: 1.69–3.53, p < 0.01)], even when pre-pandemic measure of weight and obesity were accounted for. In addition, reporting eating less in the first few months of the pandemic was significantly associated with weight loss in Nov/Dec 2020 (−1.41 kg, 95% CI: −2.23 to −0.59, p < 0.01), but not with the risk of obesity.

Despite no significant association of changes in sleeping behaviour with weight, sleeping more significantly increased the risk of obesity. The odds of being with obesity among those who reported more sleep compared to those who reported the same sleep was 1.55 (95% CI: 1.04–2.32, p = 0.03) in June/July 2020 and 1.54 (95% CI: 1.02–2.32, p = 0.04) in Nov/Dec 2020.

Finally, drinking more alcohol was significantly associated with higher weights in both June/July 2020 (0.74 kg, 95% CI: 0.16–1.32, p = 0.01) and Nov/Dec 2020 (0.84 kg, 95% CI: 0.24–1.44, p < 0.01) but not with obesity.

### Association of sociodemographic factors with weight and obesity

According to the results of fully adjusted models, some of the sociodemographic factors were significant predictors of older adults’ weight or obesity during the COVID-19 pandemic.

Across models with health behaviours included separately, significantly lower weights were found in women compared to men (range −1.77 to −1.58 kg in June/July 2020; −1.57 to −1.40 kg in Nov/Dec 2020). Having a university degree and above was associated with lower weight at both timepoints compared to those with lower education attainment (range −0.61 to −0.73 kg in June/July 2020; −0.53 to −0.65 kg in Nov/Dec 2020). Having low social contacts was associated with a higher weight only in June/July 2020 (range 0.90–0.98 kg). Those shielding (self-isolation or staying at home) reported higher weight only in Nov/Dec 2020 (range 0.79–0.87 kg) than those not shielding. Regarding obesity, older adults in the highest two wealth quintiles had significantly lower odds of obesity at two COVID-19 timepoints, compared to those in the lowest wealth quintile (4th wealth quintile: OR = 0.60 in June/July and 0.58 in Nov/Dec 2020 approximately; 5th wealth quintile: OR = 0.36 in June/July and 0.38 in Nov/Dec 2020 approximately).

Furthermore, results suggested that self-reported good sleep in June/July 2020 was associated with lower weights (range −0.80 to −0.66 kg in June/July 2020; −0.73 to −0.60 kg in Nov/Dec 2020) and lower risk of obesity (OR = 0.64–0.72 in June/July; around 0.70 in Nov/Dec 2020) significantly at two timepoints during the pandemic, compared to those reporting poor or fair sleep.

### Robustness checks

All analyses were repeated considering BMI as a continuous indicator, BMI categories (normal weight/ overweight/ with obesity), as well as weight change in percentage with reference to pre-pandemic weight as outcomes. Results were broadly consistent with those found for weight and obesity and can be found in Supplementary Figs. S1–S4.

## Discussion

Using a large nationally representative sample of older people living in private households in England, we explored perceived changes in health behaviours during the first months of the COVID-19 pandemic in relation to weight and obesity. We found that a considerable number of people were engaged in more unhealthy behaviours during the lockdown, such as less physical activity, more sitting and watching TV, eating more, drinking more alcohol, and sleeping more and that such changes were in turn related to higher weight and higher risks of obesity in both short run and long run.

Our results are in line with a few studies conducted on older people showing the correlation between less healthy behaviours and a higher possibility of obesity or weight gain during the COVID-19 lockdown. In northern Italy after a one-month lockdown, less physical exercise and eating more were found to predict a higher weight gain in a sample of 150 adults (mean age 48 years)^16^. In the first 6 weeks of confinement in Belgium, the self-reported weight gain of adults aged 55 years and above was associated with physical activity, consumption of snacks, or alcohol consumption^28^. Less exercise doubled the risk of weight gain, and more consumption of snacks was associated with nearly three times higher odds of weight gain. Older people consuming more alcohol were twice more likely to gain weight^28^. Our results are also in line with studies on a wider age group. For example, according to a survey conducted one month after the Lithuanian lockdown, weight gain in adults aged at least 18 was attributed to less exercise, drinking more alcohol, as well as eating more than usual^15^. After 2-month confinement at home in Northern Italy, more sedentary behaviours of over 2800 students and over 800 academic workers were reported to result in a larger increase in weight, 0.4 kg compared to 0.3 kg in those with fewer sedentary behaviours^29^. Decreased length of sleep was found to be one predictor of weight gain during self-quarantine in the US. This was explained by the correlation between less sleep and more snacking after dinner which led to weight gain^30^.

Compared to existing evidence on the effect of changes in health behaviours on weight gain, we contributed by focusing on older people’s obesity. Furthermore, individual weight or obesity status before the pandemic was controlled for, which was not available in most studies in the setting of the COVID-19 pandemic^15,16,28,30^. Apart from immediate effects on weight and obesity, we also reported persistent effects over time. We found that health behaviour changes in the first months of the lockdown related to weight and obesity not only in a short but also in a longer period of time (as long as 6 months). This finding is consistent with the literature revealing the association of dietary and lifestyle changes such as physical activity, alcohol intake, sleep duration and TV watching, with long-term weight gain^42,43^.

Furthermore, in our study, protective effects of some healthy behaviours against obesity were found in Nov/Dec 2020, such as less time spent sitting, eating less and less alcohol consumption. Less time spent on sedentary behaviours usually indicated more energy expenditure which was directly related to weight loss. In addition, it could be partly due to more physical activity, which contributed to weight loss. In European adults aged 65 years old and above, participating in physical activity at least 5 days a week was associated with a 42% lower risk of obesity^24^. Eating less might be a result of different factors, such as loss of appetite or taste, feeling upset during lockdown, or weight management purposes^44^. During the lockdown, the closure of restaurants, bars and shops could have reduced the availability of alcohol, resulting in reduced alcohol consumption, especially among some older adults who were unable to place online orders^28,45^. Less alcohol consumption has been widely acknowledged as one way to manage weight since alcohol contains energy in a concentrated form and may encourage eating^46^.

Our findings also implied that weight management during the COVID-19 pandemic was more critical in certain subgroups of the population, such as less educated and poorer groups, consistent with several studies in the literature^3,33,47^. For example, lower education was associated with fewer behaviours protective against weight gain, and household income predicted a lower reduction in physical activity behaviours which benefited weight management^33^. This study supports the significance of sleep quality in affecting weight and obesity in the older population aged 50 and above^3,24^. In addition, social isolation was detrimental to weight management. It is possible that having fewer social contacts leads to less physical activity, more sedentariness, or unhealthy eating patterns^48^.

To the best of our knowledge, this study is the first attempt to investigate the association between changes in health behaviours and weight or obesity in older people living in private households in England during the COVID-19 pandemic, with pre-pandemic weight or obesity controlled. It provides novel evidence using a nationally representative sample. In addition, we are able to analyse the immediate and long-term impact of changes in health behaviours on weight and obesity during the UK lockdown. Another strength is that we investigate a wide range of health behaviours and the impact of changes in these health behaviours is analysed separately. It is more reasonable because there are usually parallel changes in health behaviours. For example, more sedentary behaviours came along with less exercise^49^, and watching TV was reported to be associated with eating snacks^50^. This study also provides additional evidence on the vulnerability of people in disadvantaged socioeconomic status, those reporting social isolation or poor sleep, in relation to weight management during the pandemic.

However, this study has some limitations. First, the accurate pre-pandemic weight is not available. Individual weight or obesity before the pandemic is measured almost 2 years before the beginning of the pandemic in 2018/19, and might differ from the weight or obesity status just before the national UK lockdown starting in March 2020. Second, during the COVID-19 pandemic, it was not possible to carry out in-person interviews. Therefore, individual weight and height in the COVID-19 sub-study are self-reported, which is less accurate compared to objective measurements. There might also be measurement errors due to the existence of round numbers in some cases. Third, more details of health behavioural changes are not collected. For instance, it is unknown what “more” or “fewer” health behaviours mean exactly as perceived changes are based on personal perceptions and can mean different things to different people.

## Conclusions and policy recommendations

To conclude we showed that during the COVID-19 pandemic in 2020, less physical activity, more sedentariness, eating more and drinking alcohol more than usual were associated with higher weight and obesity. Compared to the situation after the first UK lockdown, the association of risky health behaviours with weight and obesity was more pronounced during the second lockdown in Nov/Dec 2020. In addition, less sedentariness and eating less significantly reduced weight in Nov/Dec 2020, reflecting a long-term protective effect of healthy behaviours.

Our findings provide several policy implications. Considering potential health risks associated with weight gain and obesity, especially in older people, it is necessary for policymakers to improve weight management interventions nationwide. However, during the COVID-19 pandemic, people’s daily life experienced unanticipated changes which might sustain even after national restrictions were lifted. Therefore, it becomes more challenging for the older population to manage weight or for the government to deliver weight management programmes^48^. Under this context, national weight management intervention can be improved based on the evidence produced in this study. For example, indoor exercise guidance should be provided, with a range of moderate to vigorous physical activity options that could be useful for older people, with a particular focus on older adults with lower socioeconomic status or low social contacts.

### Supplementary Information

Supplementary Information.

## Data Availability

The datasets analysed during the current study are available in the UK Data Service (SN 8688 and 5050) repository, https://beta.ukdataservice.ac.uk/datacatalogue/studies/study?id=8688 and https://beta.ukdataservice.ac.uk/datacatalogue/studies/study?id=5050.
