# Supplementary material for: Changes in health behaviours during the COVID-19 pandemic and effect on weight and obesity among older people in England
Source: Sci Rep. 2023 Sep 5;13:14661. doi: 10.1038/s41598-023-41391-z (PMC10480155; doi:10.1038/s41598-023-41391-z)
Supplement: Supplementary file 1 — Supplementary Information. [file 41598_2023_41391_MOESM1_ESM.docx]

**Table S1 Distribution of BMI category during pandemic over pre-pandemic BMI category (N=4 182)**

| Pre-pandemic  BMI category | COVID-19 sub-study 1  in June/July 2020 | | | COVID-19 sub-study 2  in Nov/Dec 2020 | | |
| --- | --- | --- | --- | --- | --- | --- |
|  | Normal weight  % | Overweight  % | With obesity  % | Normal weight  % | Overweight  % | With obesity  % |
| Normal weight | 90.92 | 8.85 | 0.23 | 90.77 | 9 | 0.23 |
| Overweight | 19.04 | 75.45 | 5.51 | 19.62 | 74.75 | 5.63 |
| With obesity | 1.32 | 20.32 | 78.36 | 1.41 | 20.67 | 77.92 |


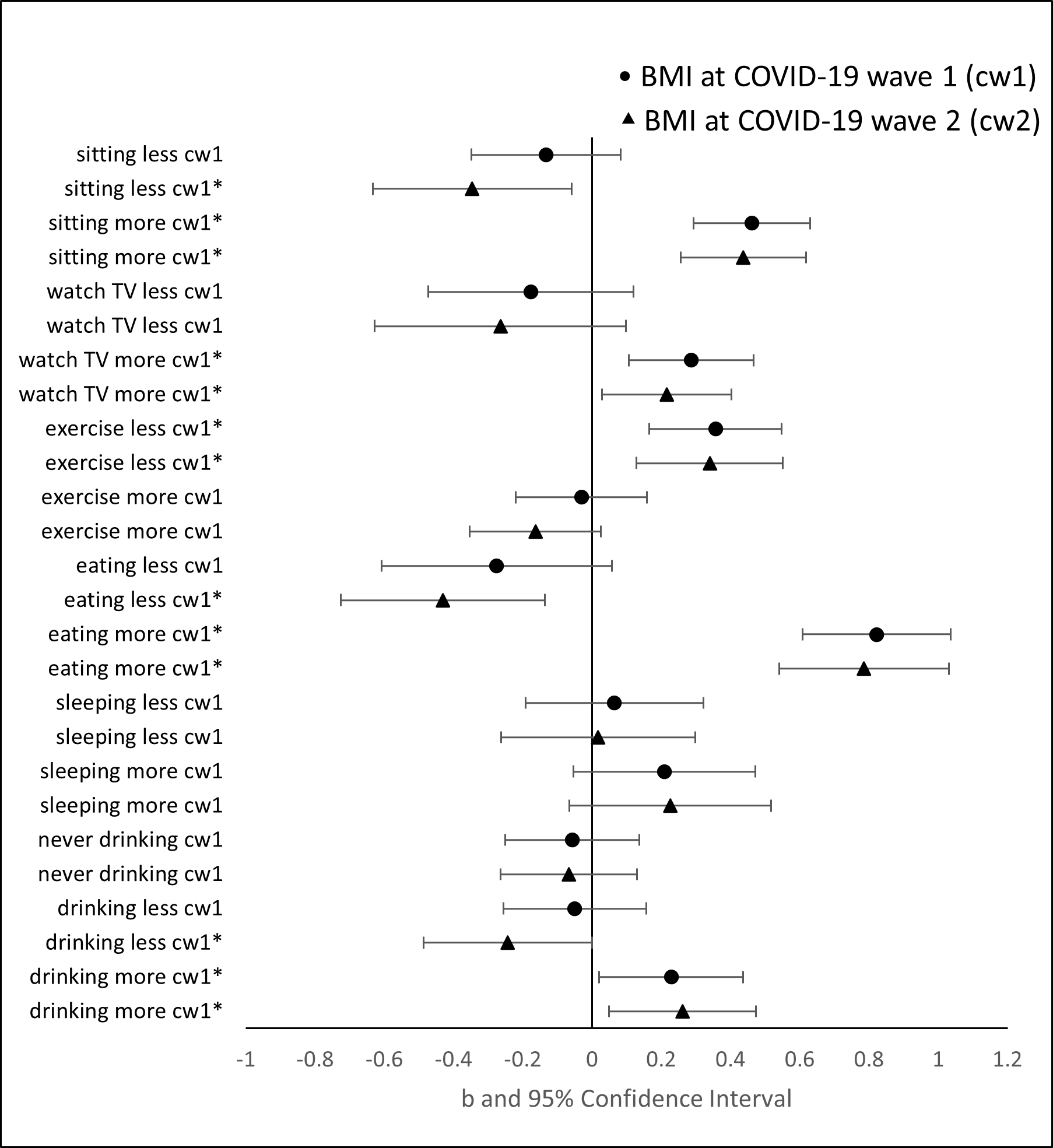


**Figure S1 Fully adjusted associations of perceived changes in health behaviours with BMI at two timepoints**

Notes: N=4 182. A continuous variable *BMI*, is used as outcome. Separate linear regression models are estimated for each health behaviour with full adjustment. The reference category of each perceived change in health behaviour is “About the same”. All models show high goodness of fit (p<0.0000). Perceived changes in health behaviours are collected in COVID-19 wave 1. The outcome of BMI is measured in two COVID-19 waves. cw1 and cw2 denote the timepoint at which variable is collected. cw1: COVID-19 wave 1 in June/July 2020; cw2: COVID-19 wave 2 in Nov/Dec 2020. *Significant at 95% confidence level.


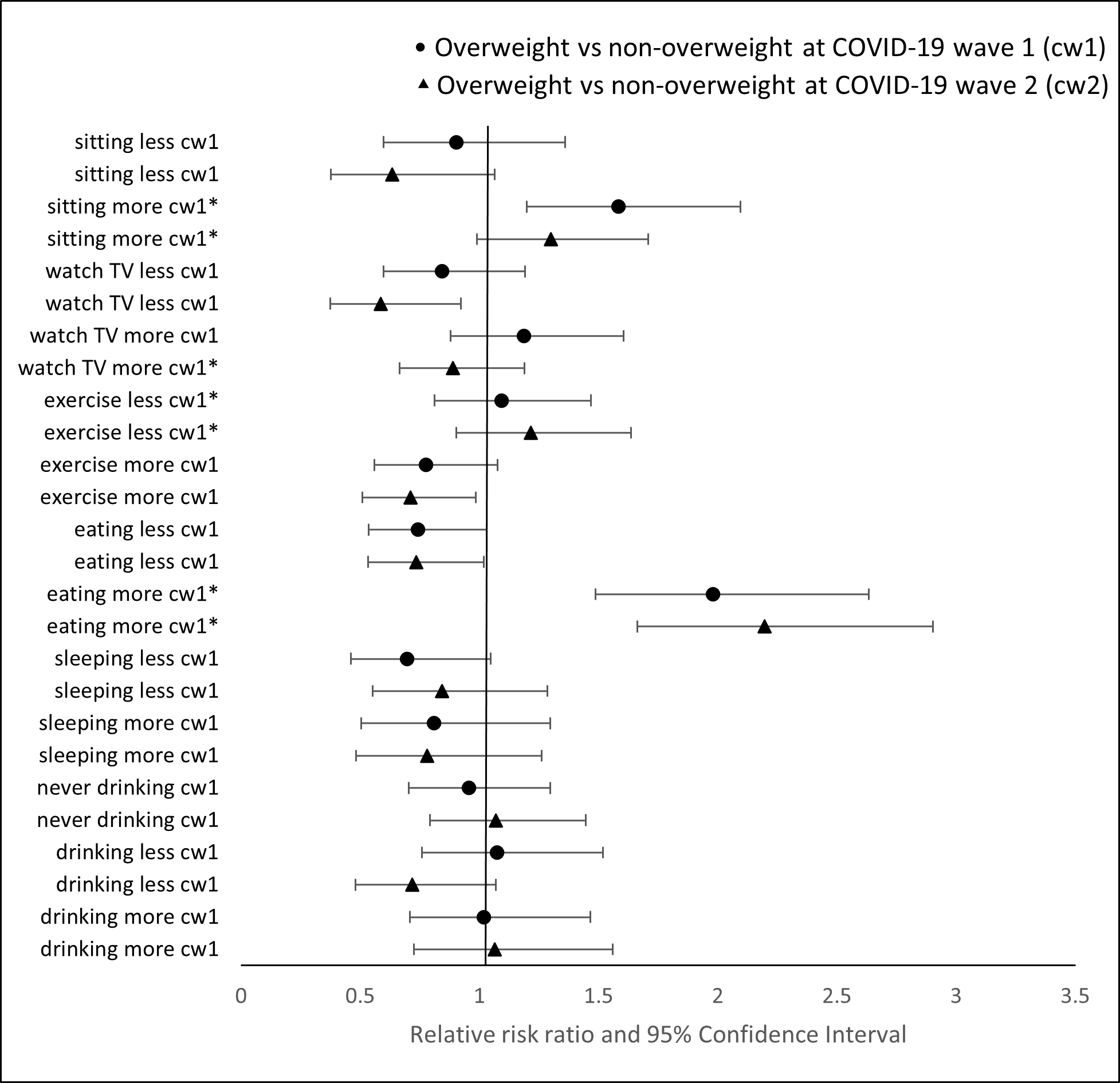


**Figure S2 Fully adjusted associations of perceived changes in health behaviours with overweight from ordinal logistic models**

Notes: N=4 182. Separate ordinal logistic regression models are estimated for each health behaviour with full adjustment. The reference category of each perceived change in health behaviour is “About the same”. In this forest plot, outcome is being overweight (BMI≥25 kg/m^2^) versus not (BMI<25 kg/m^2^). All models show high goodness of fit (p<0.0000). Perceived changes in health behaviours are collected in COVID-19 wave 1. The outcome of BMI category is measured in two COVID-19 waves. cw1 and cw2 denote the timepoint at which variable is collected. cw1: COVID-19 wave 1 in June/July 2020; cw2: COVID-19 wave 2 in Nov/Dec 2020. *Significant at 95% confidence level.


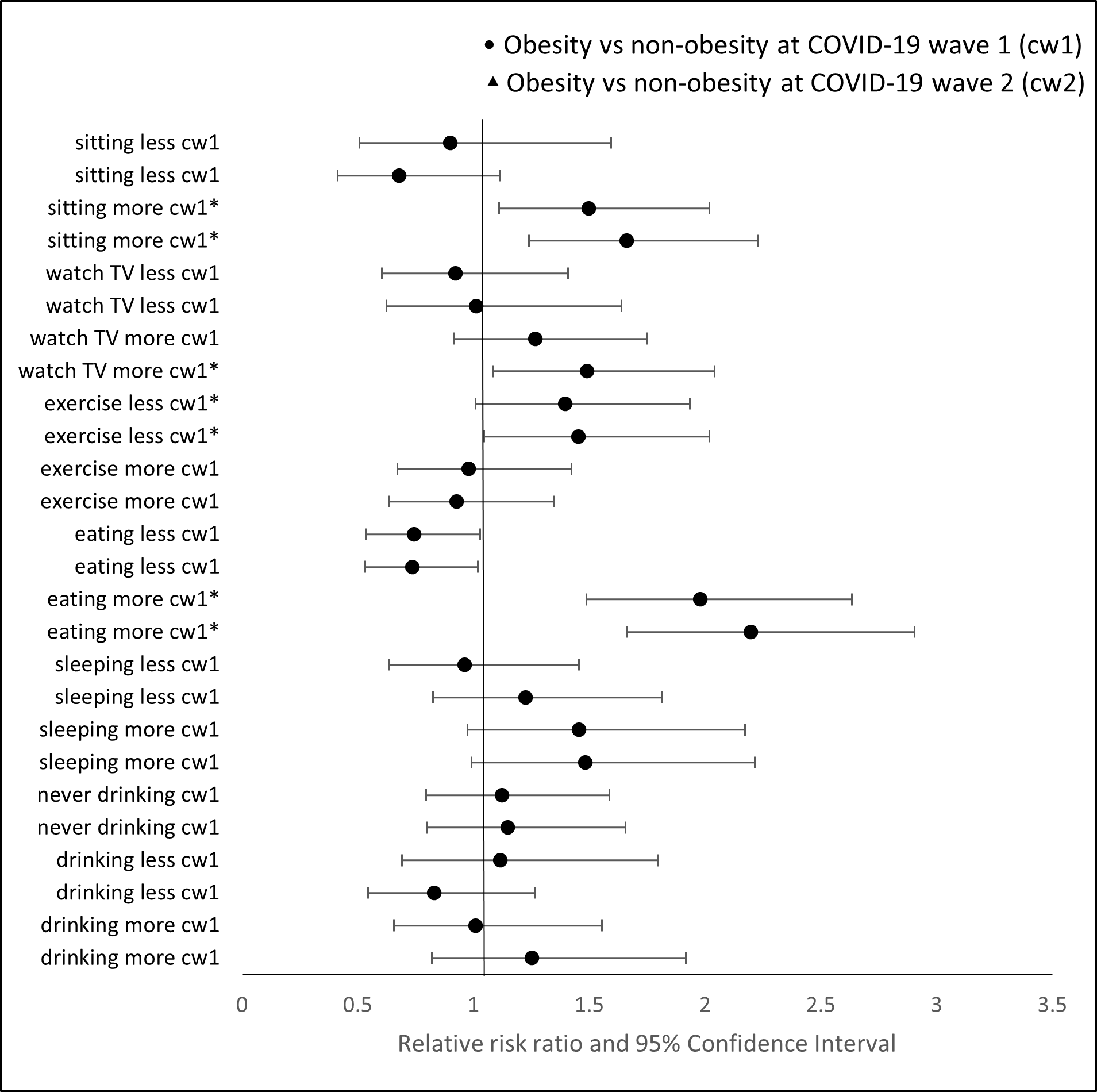


**Figure S3 Fully adjusted associations of perceived changes in health behaviours with obesity from ordinal logistic models**

Notes: N=4 182. Separate ordinal logistic regression models are estimated for each health behaviour with full adjustment. The reference category of each perceived change in health behaviour is “About the same”. In this forest plot, outcome is obesity (BMI≥30 kg/m^2^) versus not (BMI<30 kg/m^2^). All models show high goodness of fit (p<0.0000). Perceived changes in health behaviours are collected in COVID-19 wave 1. The outcome of BMI category is measured in two COVID-19 waves. cw1 and cw2 denote the timepoint at which variable is collected. cw1: COVID-19 wave 1 in June/July 2020; cw2: COVID-19 wave 2 in Nov/Dec 2020. *Significant at 95% confidence level.


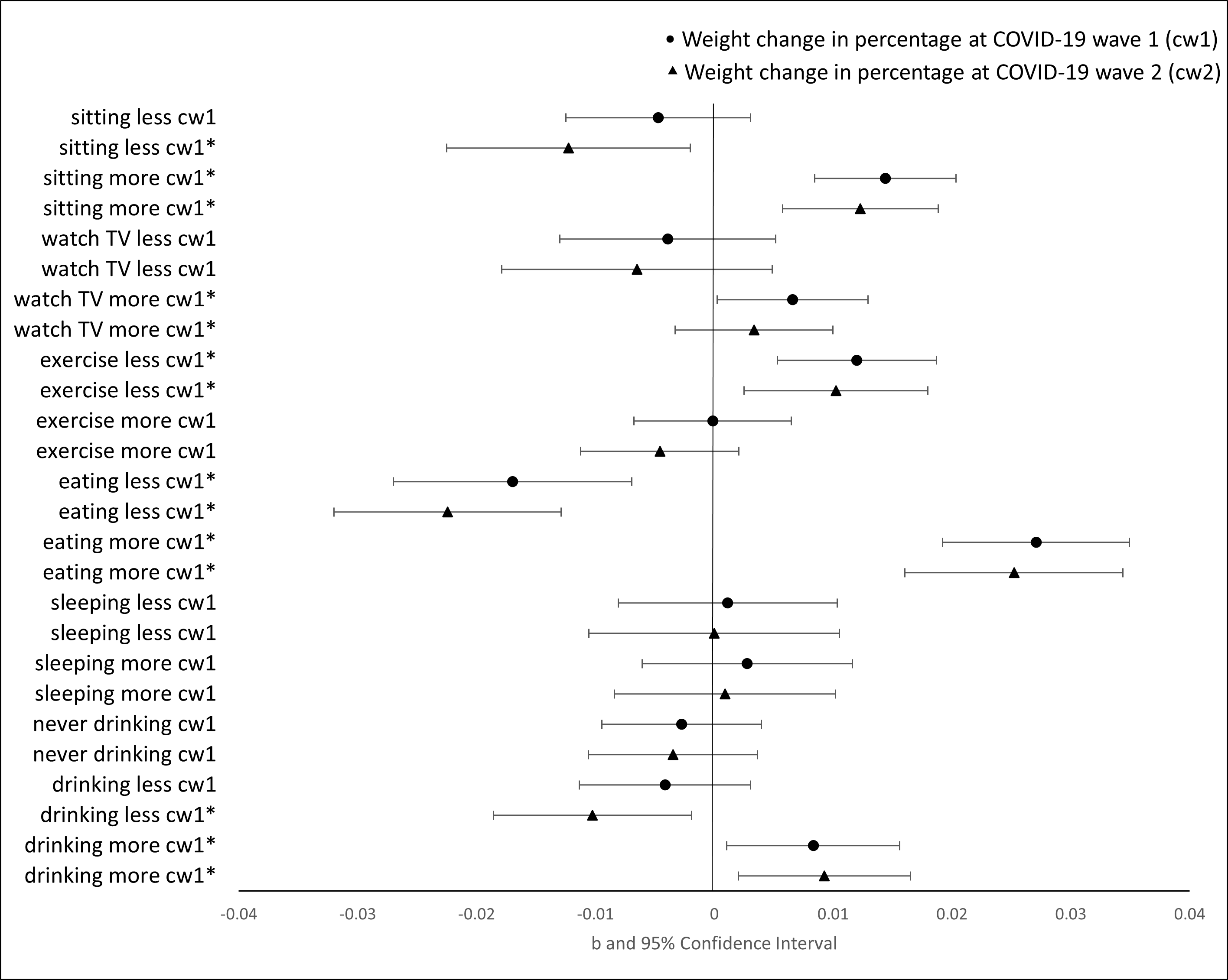


**Figure S4 Fully adjusted associations of perceived changes in health behaviours with weight change in percentage**

Notes: N=4 182. A continuous variable *weight change in percentage*, is used as outcome. Separate linear regression models are estimated for each health behaviour with full adjustment. The reference category of each perceived change in health behaviour is “About the same”. All models show high goodness of fit (p<0.0000). Perceived changes in health behaviours are collected in COVID-19 wave 1. The outcome of weight change in percentage is measured in two COVID-19 waves. cw1 and cw2 denote the timepoint at which variable is collected. cw1: COVID-19 wave 1 in June/July 2020; cw2: COVID-19 wave 2 in Nov/Dec 2020. *Significant at 95% confidence level.
